# Supplementary material for: Identification of photoperiod-induced specific miRNAs in the adrenal glands of Sunite sheep (Ovis aries)
Source: Front Vet Sci. 2022 Jul 22;9:888207. doi: 10.3389/fvets.2022.888207 (PMC9354845; doi:10.3389/fvets.2022.888207)
Supplement: Supplementary file 1 [file Data_Sheet_1.ZIP › Supplementary file/miRDeep2 file.zip!/miRDeep2 file/Novel_Structure/SP42A2/Novel_259.pdf]

Provisional ID	:	NC_019469.2_24414
Score total	:	42.6
Score for star read(s)	:	3.9
Score for read counts	:	36.3
Score for mfe	:	0.8
Score for randfold	:	1.6
Score for cons. seed	:	
Total read count	:	83
Mature read count	:	80
Loop read count	:	0
Star read count	:	3

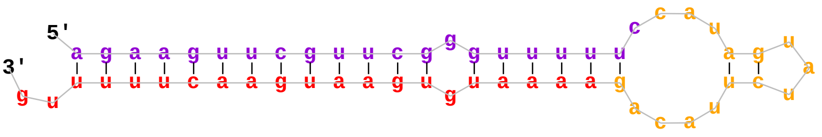

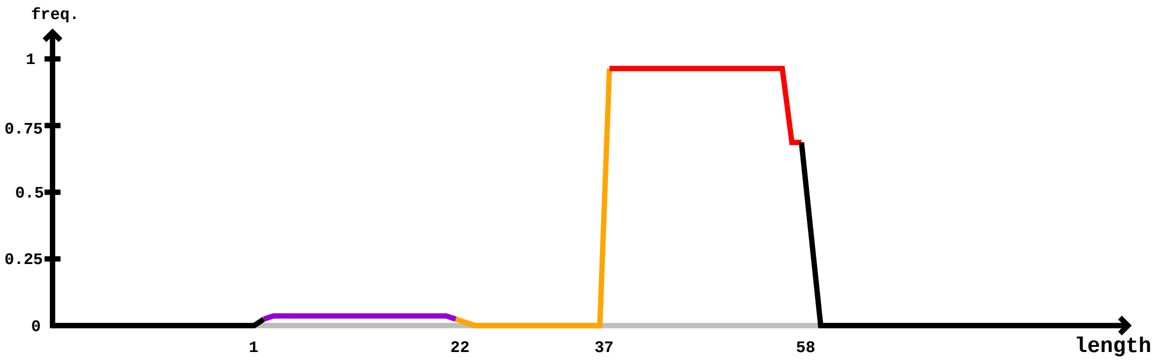

Star Mature

```
5'- uugcuguauuggguuggcccagaaguucguucggguuuuuccauaguaucuuacagaaaaugugaaugaacuuuuuggcccacacaacacucugauaauuuucccacuua  -3'  obs
    uugcuguauuggguuggcccagaaguucguucggguuuuuccauaguaucuuacagaaaaugugaaugaacuuuuuggcccacacaacacucugauaauuuucccacuua        exp
    .....((.(((.((.((((.(((((((((((((.((((((....((...))....)))))).))))))))))))).)))).)).))).))....................   reads  mm  sample
    ....................agaaguucguucggguuuuu......................................................................   1      0   seq
    ....................agaaguucguucggguuuuuc.....................................................................   1      0   seq
    .....................gaaguucguucggguuuuuc.....................................................................   1      0   seq
    ........................................................aaaauCugaaugaacuuuu...................................   23     1   seq
    ........................................................aaaauCugaaugaacuuuuug.................................   57     1   seq
```
